# Genetic diversity analysis of Chinese *Leishmania* isolates and development of *L. donovani* complex-specific markers by RAPD

**DOI:** 10.1186/s12879-021-06163-y

**Published:** 2021-05-21

**Authors:** Dongmei Yuan, Hanxiao Qin, Dali Chen, Jianping Chen

**Affiliations:** 1 Department of Human Anatomy, West China School of Basic Medical Sciences and Forensic Medicine, Sichuan University, Chengdu, 610041 China; 2 Department of Parasitology, West China School of Basic Medical Sciences and Forensic Medicine, Sichuan University, Chengdu, 610041 China; 3 Animal Disease Prevention and Food Safety Key Laboratory of Sichuan Province, Sichuan University, No.17 People’s South Road, Chengdu, Sichuan Province China

**Keywords:** *Leishmania donovani*, China, RAPD, SCAR

## Abstract

**Background:**

Leishmaniasis is one of the most neglected tropical diseases in the world and remains endemic in some underdeveloped regions, including western China. The phylogeny and classification of Chinese *Leishmania* has not been completely clarified to date, especially within the *Leishmania* (*L.*) *donovani* complex, although phylogenetic analyses based on a series of gene markers have been performed. More analytic methods and data are still needed. Random amplified polymorphic DNA (RAPD) technology can sensitively identify slight intraspecific differences, and it is a powerful tool to seek species-specific markers. This work attempted to identify Chinese *Leishmania* isolates from diverse geographic regions at the genomic level. Meanwhile, specific markers of the *L. donovani* complex were also developed by RAPD.

**Methods:**

RAPD was applied to 14 Chinese *Leishmania* isolates from diverse geographic regions and 3 WHO reference strains. The polymorphic sites of amplification were transformed into a data matrix, based on which genetic similarity was calculated, and a UPGMA dendrogram was constructed to analyse the genetic diversity of these *Leishmania* isolates. Meanwhile, the specific amplification loci of the *L. donovani* complex were TA-cloned, sequenced and converted into sequence characterized amplified region (SCAR) markers, which were validated preliminarily in 17 available *Leishmania* strains in this study and analysed by bioinformatics.

**Results:**

The cluster analyses showed that the three *Leishmania* sp. isolates SC10H2, SD and GL clustered together and apart from others, the strains of the *L. donovani* complex clearly divided into two clades, and the three isolates Cy, WenChuan and 801 formed a subclade. Three specific SCAR markers of the *L. donovani* complex, i.e., 1-AD17, 2-A816 and 3-O13, were successfully obtained and validated on 17 available *Leishmania* strains in this study. Through bioinformatic analyses, Marker 1-AD17 may have more specificity for PCR detection of VL, and Marker 3-O13 has the potential to encode a protein.

**Conclusions:**

The RAPD results verified that the undescribed *Leishmania* species causing visceral leishmaniasis (VL) in China was a unique clade distinguished from *L. donovani* and revealed that there was genetic differentiation among Chinese *L. donovani*. The identification of *L. donovani*-specific markers may help to provide a foundation for future research attempting to develop new specific diagnostic markers of VL and identify specific gene functions.

**Supplementary Information:**

The online version contains supplementary material available at 10.1186/s12879-021-06163-y.

## Background

Leishmaniasis is a tropical disease caused by the obligate intracellular protozoan genus *Leishmania* and is transmitted through bites of the genus *Phlebotomus*, which threatens 350 million people over 98 countries, primarily in developing countries [1]. There are four main forms of leishmaniasis according to different clinical syndromes, i.e., visceral leishmaniasis (VL, kala-azar), post-kala-azar dermal leishmaniasis (PKDL), cutaneous leishmaniasis (CL) and mucocutaneous leishmaniasis (MCL), which are caused by different species of *Leishmania*. There are more than 64 species of the genus *Leishmania* consisting of the subgenera *Euleishmania*, *Paraleishmania* and *Sauroleishmania* [2], among which 20 species are considered infectious to humans [1].

Visceral leishmaniasis, which is acute and fatal if left untreated, is the main form of leishmaniasis that prevails in China. Although Chinese VL has been effectively restricted since the 1950s, there are still localized and sporadic outbreaks now, mostly in the Xinjiang Uygur Autonomous Region, Sichuan, and Gansu province [3]. Moreover, China is still one of 14 high-burden VL countries [4]. Over the past few decades, epidemiological characteristics, kinetoplasts and chromosomal DNA have been applied successively to Chinese VL typing [5–7]. In recent years, a series of gene markers, such as internal transcribed spacer 1 (ITS1), cytochrome oxidase II (COX II), cytochrome *b* (cyt *b*) and HSP70, have been applied to establish phylogenetic trees, and the *L. donovani* complex (including *L. donovani* and *L. infantum*), *Leishmania gerbilli*, *Leishmania tropica* and *Leishmania turanica* have been identified, along with an undescribed *Leishmania* species that has clustered with lizard *Leishmania* [8–11]. However, the species classification and pathogen identification of Chinese leishmaniasis is far from complete, especially within the *L. donovani* complex. Our previous phylogenetic analyses on HSP70 indicated a clear relationship within the *L. donovani* complex [11]. This finding indicated that *L. infantum*, which is one of the causative agents of VL, is primarily distributed in western mountainous areas and plains of northwestern China, including Sichuan, Gansu, and Xinjiang provinces. The identification of MHOM/CN/80/801 isolates from VL patients in Kashi, Xinjiang, was different than the results of a study using ITS1 sequences [12]. Moreover, the analysis of HSP70 concluded that *L. donovani* is the pathogen of CL in Karamay of Xinjian, and *Phlebotomus major wui* is the vector, which challenges the previous determination that *L. infantum* is the pathogen of CL in Karamay based on gene hybridization and animal inoculation [13, 14]. Thus, the application of more diverse analytical methods and more classified data is needed to deepen our knowledge of the genetic relationship of Chinese *Leishmania* isolates.

Rapid species identification is essential for the early diagnosis of leishmaniasis and is conducive to accurate treatments. Multilocus enzyme electrophoresis (MLEE) is still the golden standard in the identification of *Leishmania* species [15], but it is rarely used now because of its time-consuming procedure. DNA markers have been widely applied for phylogenetic research in *Leishmania* [16]. The phylogenetic trees of these markers provide much evidence for the taxonomy of the main *Leishmania* complex, but the relatively slow evolutionary rates of these genes are insufficient to solve the species relationship within the complex [17]. Furthermore, the discrimination capability among markers is diverse, which in turn makes the identification of species and subspecies sometimes inconsistent [18]. Random amplification polymorphic DNA (RAPD) is a technique that can be used for polymorphic analysis of unknown genomes on the basis of PCR [19, 20]. This technique is easy and sensitive and has always been applied for species identification [21] and correlation analysis of population differentiation with geographical origins in *Leishmania* [22]. Additionally, RAPD has advantages in taxonomy at the subgeneric level [23] and species level [24]. Meanwhile, through the selection of DNA markers among differentially amplified bands, the specific genetic markers of one species can be developed to perform species identification or assist with diagnosis via the creation of a probe. Therefore, RAPD has proved to be an effective method to obtain genetic markers for the development of relevant *Leishmania* DNA assays [25, 26].

In this study, RAPD was applied to Chinese *Leishmania* isolates from diverse geographic regions, which could help us more thoroughly understand the genetic differences of these isolates, especially the *L. donovani* complex*.* Meanwhile, the specific amplified bands of the *L. donovani* complex were screened and converted into *L. donovani* complex specific sequence characterized amplified regions (SCAR) markers. These SCAR markers were validated preliminarily in 17 available *Leishmania* strains in this study and analysed by bioinformatics, which may provide a foundation for research on specific gene functions and the development of new diagnostic markers of VL.

## Methods

### Leishmania strains and DNA extraction

Fourteen Chinese *Leishmania* isolates and three WHO reference strains were used in this study and are listed in Table 1. The 14 Chinese *Leishmania* isolates were collected from plain, hill, and desert foci in China. These parasites were cultured in Medium 199 supplemented with 15% heat-inactivated foetal bovine serum, 100 U/mL penicillin (Sigma) and 100 μg/mL streptomycin (Sigma) at 26 °C. The promastigotes were collected at logarithmic phase and centrifuged at 3300×g for 10 min. Total DNA was extracted using a commercially available DNA extraction kit (TianGen Cell DNA Kit). The concentrations of 17 DNA samples were detected (Thermo Scientific™ NanoDrop™ One) and adjusted to the same level before subsequent RAPD amplification.

**Table 1:** Detailed information of *Leishmania* strains that were used for RAPD analyses in this study

Name	Species	WHO code	Disease type	Location	Host
KXG-918	*L. donovani*	IMJW/CN/91/KXG-918		Karamay, Xinjiang, China	*Phlebotomus major wui*
KXG-65	*L. donovani*	IMJW/CN/87/KXG-65		Karamay, Xinjiang, China	*Phlebotomus major wui*
9044	*L. donovani*	MHOM/CN/90/9044	VL	Shandong, China	*Homo sapiens*
KXG-927	*L. donovani*	IMJW/CN/92/KXG-927		Karamay, Xinjiang, China	*Phlebotomus major wui*
SC6	*L. donovani*	MHOM/CN/86/SC6	VL	Nanping, Sichuan, China	*Homo sapiens*
801	*L. donovani* ^a^	MHOM/CN/80/801	VL	Kashgar, Xinjiang, China	*Homo sapiens*
DD8	*L. donovani*	MHOM/IN/80/DD8^b^	VL	India	
KXG-XU	*L. infantum* ^a^	MHOM/CN/93/KXG-XU	CL	Karamay, Xinjiang, China	*Homo sapiens*
KXG-LIU	*L. infantum* ^a^	MHOM/CN/94/KXG-LIU	CL	Karamay, Xinjiang, China	*Homo sapiens*
Cy	*L. infantum*	MCAN/CN/08/Cy	CanL	Wudu, Gansu, China	*Canis lupus familiaris*
WenChuan	*L. infantum*	MCAN/CN/90/WenChuan	CanL	Wenchuan, Sichuan, China	*Canis lupus familiaris*
EJNI-154	*L. gerbilli*	MRHO/CN/81/EJNI-154		Ejina, Inner Mongolia, China	*Rhombomys opimus*
GERBILLI	*L. gerbilli*	MRHO/CN/60/GERBILLI^b^		Gansu, China	*Rhombomys opimus*
K27	*L. tropica*	MHOM/SU/74/K27^b^			
SD	*Leishmania* sp.		VL	Shandong, China	*Homo sapiens*
GL	*Leishmania* sp.		VL	Gansu, China	*Homo sapiens*
SC10H2	*Leishmania* sp.	MHOM/CN/90/SC10H2	VL	Sichuan, China	*Homo sapiens*

^a^the species of this strain is controversial based on different classification method

^b^WHO reference strain

### RAPD-PCR

Twenty decamer primers were selected according to previous studies [27, 28] and commercially synthesized (Invitrogen). All primers were prepared as 10 μM (10 pmol/μl) working solutions. These primers were first screened through three independent RAPD amplifications on DNA of the same *L. donovani* isolate, and then 10 primers that presented polymorphic, reproducible and clear amplification profiles were selected (Table 2). RAPD amplification was performed in 50 μl reactions containing 0.8 μM primer, 10 ng of genomic DNA sample, 25 μl of 2 × TaqMater Mix (Tsingke, China) and PCR-grade distilled water. The PCR procedure was as follows: initial denaturation at 94 °C for 5 min followed by 45 cycles of 94 °C for 1 min, 36 °C for 1 min, 72 °C for 2 min, and a final extension at 72 °C for 8 min. The PCR products were separated using 1.5% agarose gel electrophoresis. Each PCR and electrophoresis separation were performed three times with the same protocol and operator to assure reproducibility.

**Table 2:** The nucleotide sequences of 10 primers and amplification results of *Leishmania* isolates

Primers	Nucleotide Sequence 5′-3’	Total Bands
**OP-AD17**	GGCAAACCCT	13
**OP-AY5**	TCGCTGCGTT	12
**OP-AY8**	AGGCTTCCCT	18
**OP-O13**	GTCAGAGTCC	10
**OP-U3**	CTATGCCGAC	11
**AB1–13**	TTCCCCCGCT	12
**AB1–09**	TGGGGGACTC	12
**AB1–04**	GGACTGGAGT	7
**3301**^**35**^	TCGTAGCCAA	12
**A816**	GTGACGTAGG	14
**Total**		121

### Phenetic analysis of RAPD results

The bands of all polymorphic RAPD gels were marked as “0” for absent and “1” for present. The relative intensity among all bands was disregarded. A 0/1 data matrix was created in Microsoft Excel 2013 and analysed using the Numerical Taxonomy and Multivariate Analysis System (NTSYS) [29]. The similarity module was used to calculate the similarity matrix. The SAHN function of the clustering module was employed for clustering analysis, and the phenetic dendrogram was output under the unweighted pair-group method with arithmetic means (UPGMA).

### Cloning, sequencing and verification of SCAR markers

The *L. donovani* and *L. infantum* strain-specific bands that were stably reproduced by gel electrophoresis were considered potential SCAR markers of the *L. donovani* complex for the following extraction. These gel blocks were purified and then cloned into the pGM-T vector (Tiangen, China) overnight. Recombined products were mixed with DH5α competent *E. coli* and then screened by blue-white selection. The white colonies were picked and identified by colony PCR, and then the positive samples were cultured in liquid LB medium and collected for DNA sequencing (Tsingke, China). The specific primer pairs of the obtained SCAR marker sequences were designed using Primer 5.0.

Then, PCR was performed on 17 currently available *Leishmania* strains to verify the specificity of these markers for the *L. donovani* complex in this study. For the PCR conditions, annealing temperature was tested by gradient for different primers of each SCAR marker.

### Bioinformatic analysis of SCAR markers

The obtained SCAR markers sequences were submitted to BLAST online for homology analyses in the NCBI database, and the base component was analysed using Lasergene EditSeq. The open reading frames (ORFs) were predicted and located using NCBI-ORF Finder. To further determine whether the ORFs contained in the sequences have the potential to express proteins, the promoter binding sites were analysed and predicted online using Promotor Scan. Using Lasergene, the ORFs that demonstrated potential protein expression were translated, and the components of these presumed proteins were analysed. The secondary structures were predicted by the Chou-Fasman loading method. The hydrophobic regions were calculated using the Kyte-Doolittle method. The antigenic determinants were analysed through the JamesonWolf method, and the surface probability was assessed using the Emini method.

## Results

### RAPD analysis of 17 Leishmania strains

**Fig. 1:**
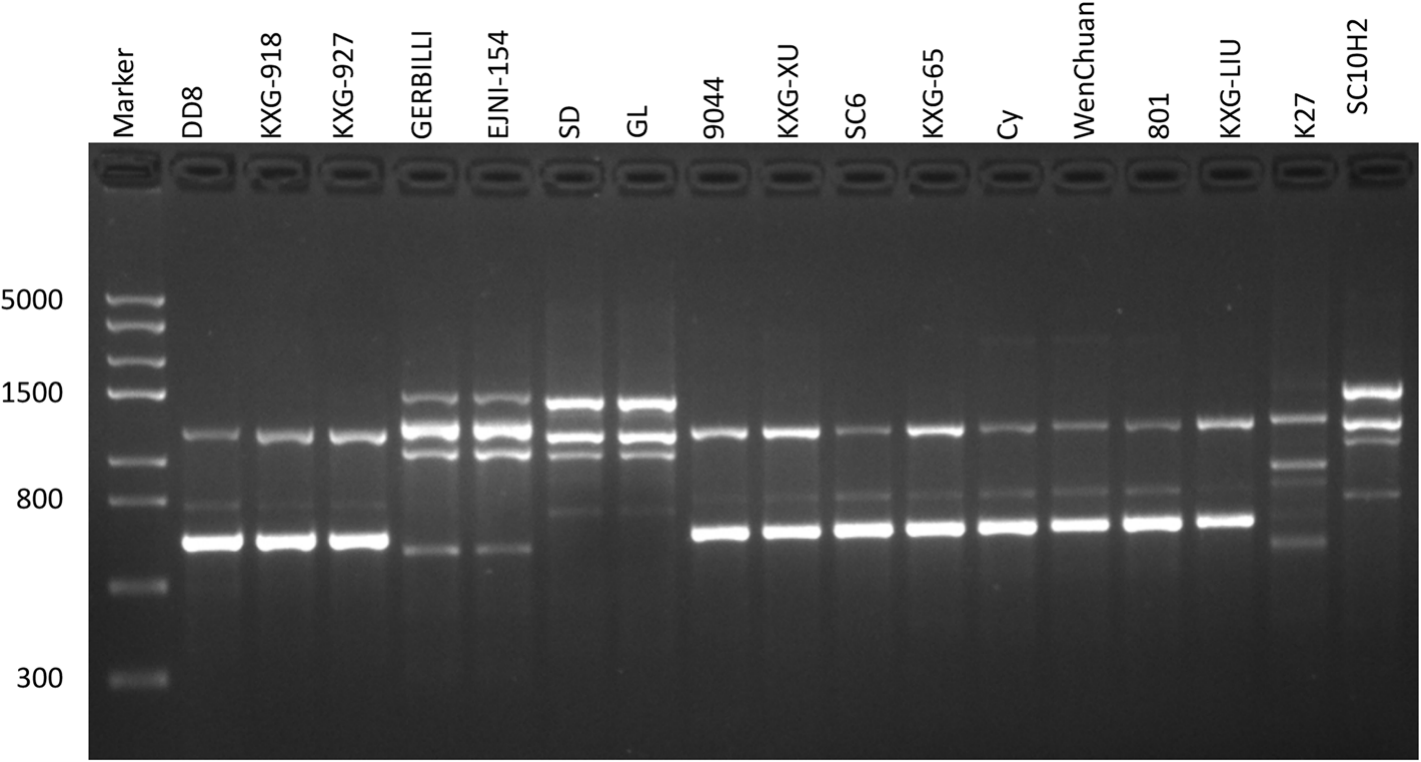
The RAPD profiles of 17 *Leishmania* strains obtained with primer OP-AD17

**Fig. 2:**
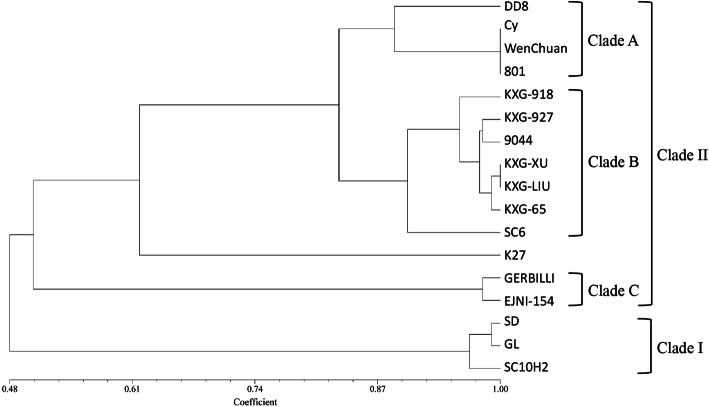
UPGMA tree of 17 *Leishmania* strains clustered based on genetic similarity

Through RAPD-PCR of the 17 *Leishmania* strains, a total of 121 RAPD bands were observed, of which 120 bands were polymorphic (99.17%). An average of 12.1 bands were amplified by each primer, and segments ranged from 200 to 3000 bp. According to the gel photographs, there were differences among the 17 *Leishmania* isolates. Figure 1 shows a gel photo with an example of polymorphism. The same species tended to form similar band models. The genetic similarity of 17 *Leishmania* strains ranged from 0.4393 to 1.0000 with an average of 0.6758 (Table S1, see Additional file 1), which indicated considerable genetic differentiation among these isolates. The UPGMA dendrogram established based on the similarity matrix is shown in Fig. 2. The isolates SC10H2, SD and GL clustered into Clade I with a high average similarity index of 0.9715, indicating a distant genetic relationship with the others. Other strains formed Clade II, which further consisted of Clades A, B and C. The isolate EJNI-154 clustered with the *L. gerbilli* WHO reference strain MRHO/CN/60/GERBILLI and formed Clade C. The strains that were previously identified as *L. donovani* complex did not cluster as one clade but two clades, Clades A and B, instead. The isolates Cy, WenChuan and 801 clustered with *L. donovani* reference strain DD8 as Clade A, whose average similarity index was 0.9439. The other *L. donovani* complex strains clustered as Clade B, in which genetic differences still existed. Within Clade B, the similarity index of SC6 from Sichuan Province with others was 0.9019, which was significantly lower than the average index of Clade B (0.9537), so SC6 was separated into an independent clade.

### Cloning, sequencing and verification of SCAR markers

**Fig. 3:**
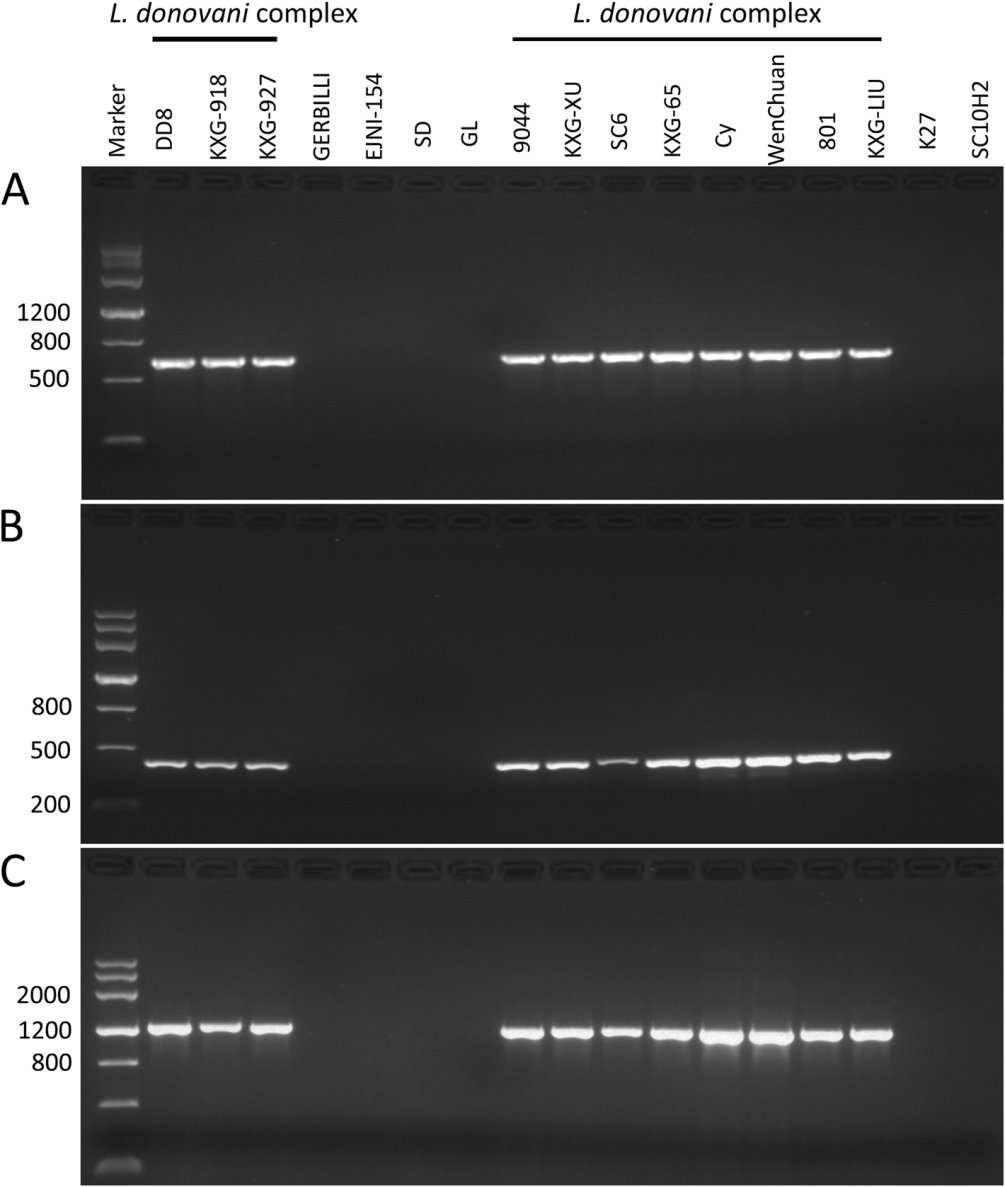
Species-specific amplification of the three SCAR markers of the *L. donovani* complex. A: 1-AD17; B: 2-A816; C: 3-O13

A total of four fragments that appeared only in all *L. donovani* complex strains were successfully T-cloned and sequenced. The obtained fragment sequences were named partly after their primers, i.e., 1-AD17, 2-A816, 3-O13 and 4–09. To evaluate the species specificity, an 18 base pair primer was designed for each potential marker. By PCR amplification of the 17 strains in this study, three of the four markers manifested strict species-specific single bands at their corresponding loci and were tentatively converted to potential SCAR markers of the *L. donovani* complex (Fig. 3). The primers and annealing temperature of the three SCAR markers are shown in Table 3. The DNA sequences are shown in Table S2 (see Additional file 2).

**Table 3:** The converted SCAR markers of the *L. donovani* complex in this study

SCAR marker	Length	Primer sequences 5′-3’	Annealing temp (°C)
**1-AD17**	636	F: TGGCAAACCCTGTATGAGGAAAACGT R: TTGGCAAACCCTCATAGGTTGC	61
**2-A816**	416	F: GTGACGTAGGCATGCCAGCAAGGTGG R: GTGACGTAGGGGTGGGGATGAAGAGG	63
**3-O13**	1210	F: GTCAGAGTCCTCGCGGGGTATTC R: GTCAGAGTCCAGTAGATAGGATCGATGCG	65

### Bioinformatic analysis of SCAR markers

**Fig. 4:**
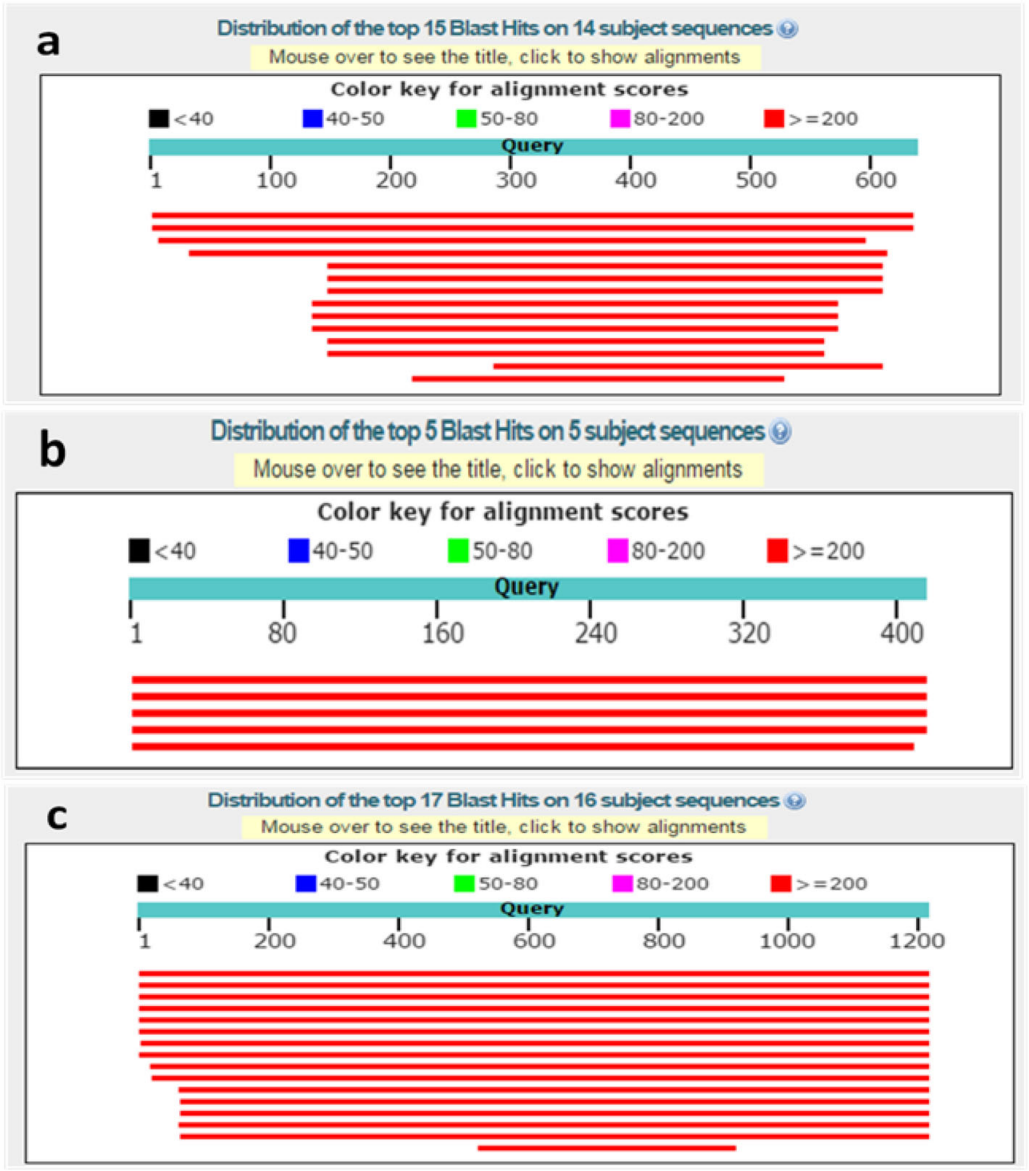
Distribution of BLAST hits of SCAR markers. a. 1-AD17; b. 2-A816; c. 3-O13

All three markers were subjected to BLAST in NCBI, the query coverage was 99%, and the identity percent was greater than 98% with *L. donovani/infantum* reference sequences. In addition, the E-valves were close to ‘0’. All results indicated that the three marker sequences had very high homology with *L. donovani/infantum* reference sequences. According to the distribution of BLAST hits of SCAR markers, except for the first two *L. donovani/infantum* reference sequences, marker 1-AD17 had a query coverage less than 90% and had no matching primer binding sites with other *Leishmania* species sequences (Fig. 4a). Markers 2-A816 and 3-O13 had more matching primer binding sites with other *Leishmania* species sequences except *L. donovani/infantum* reference sequences (Fig. 4b, c). Therefore, the primers for marker 1-AD17 have a greater specificity for amplification of the *L. donovani* complex.

**Fig. 5:**
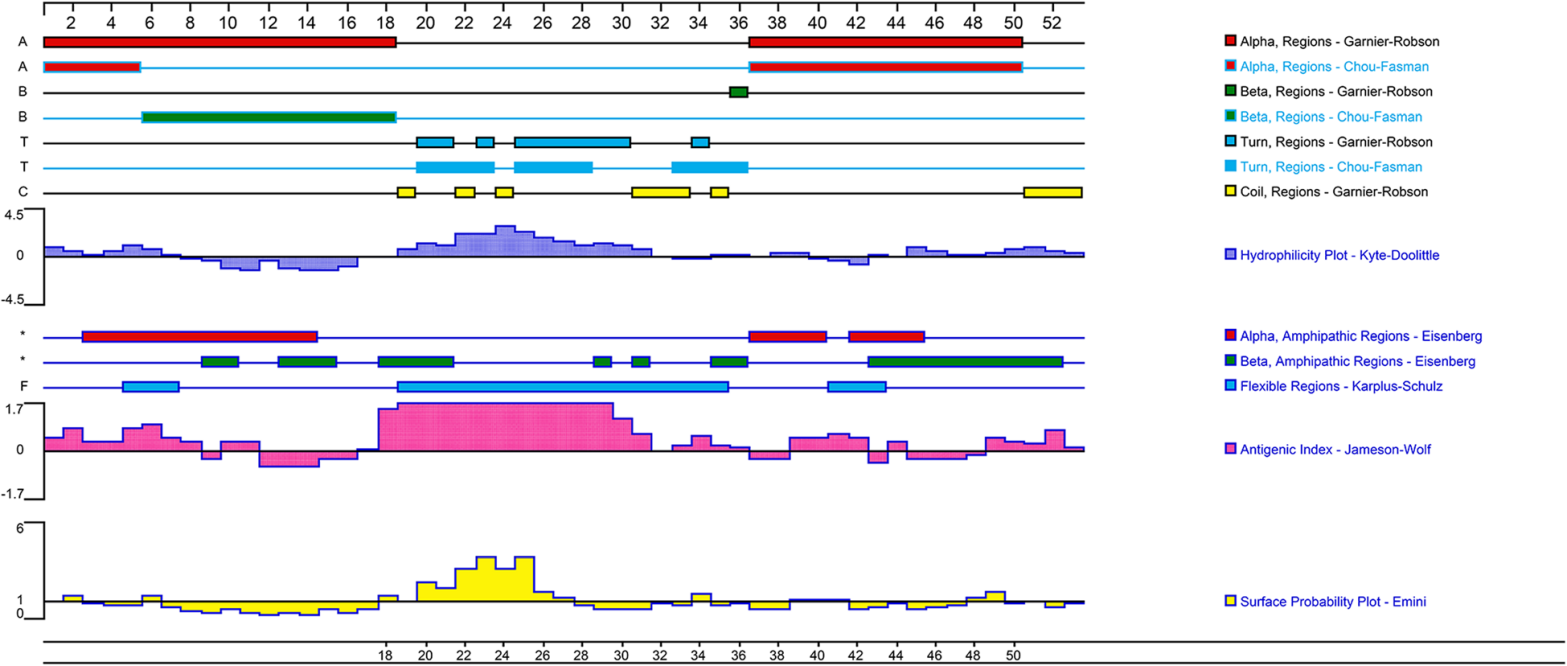
Protein predictive analytics of ORF-4 of Marker 3-O13 by Lasergene Protean

The results for sequence components, chromosomal assignments, ORFs and promotor prediction for the three markers are listed in Table 4. There were 4 to 7 ORFs in these markers, of which only 3-O13 had two potential promotors, located at 611–816 bp of the sense strand and 1056–806 bp of the antisense strand. The distance between predicted promotor sites and ORFs implied that ORF-4 has the potential to encode proteins. The gene sequence was then translated into protein and analysed by Lasergene. The protein sequence of ORF-4 contains 7 strong basic amino acids, 6 strong acid amino acids, 19 hydrophobic residues and 10 polar residues, and the putative isoelectric point is 8.835. The structural prediction of the ORF-4 protein sequence by Lasergene Protean is shown in Fig. 5. The results showed that a clear structure of one hydrophilic β turn region (19–36 residues) was flanked by two hydrophobic α helices (1–18 and 37–50 residues), which implied that the α helices might be located in the interior of the protein and that β turns might be located on the surface. This was confirmed by surface probability analysis (Emini method). In addition, the β region has a higher antigen index. Furthermore, there is no homologous protein according to BLAST in GenBank.

**Table 4:** Bioinformatic analysis of the three SCAR markers

Marker	Length	Base components				chromosomal assignment	ORFs Num.	Promotor Num.	ORF with potential of translation
		A	T	C	G				
**1-AD17**	636	130	144	165	197	22	6	0	0
		20.44%	22.64%	25.94%	30.97%				
**2-A816**	416	66	112	117	121	36	4	0	0
		15.87%	26.92%	28.12%	29.09%				
**3-O13**	1210	238	249	327	396	36	7	2	ORF-4
		19.67%	20.58%	27.02%	32.73%

## Discussion

Generally, DNA markers are now the most widely used method in the identification and classification of *Leishmania* since they are both effective and efficient. Different evolutionary rates of diverse gene markers may lead to different classification results. Thus, to help us understand interspecific relationships more comprehensively, more dissimilar identification methods would be necessary. RAPD has been widely used in genetic map construction, breeding line identification and gene marker screening and in the genetic evolution of parasites such as trypanosome, schistosome and trichinella spiralis [30–32]. As we all know, RAPD has innate drawbacks in terms of the stability and repeatability of bands due to the highly random hybrid sites with template DNA. Therefore, to obtain stable bands and repeatable results with RAPD, we used PCR amplification conditions whose stability and repeatability have been already reported [27, 28] and validated the results before formal experiments. Meanwhile, the usage of commercial Taq DNA Mix from the same batch replaced the addition of dNTPs, Mg^2+^ and Taq DNA polymerase one by one, which also improved the reaction stability in this study. Theoretically, the larger the number of RAPD polymorphic sites, the more reliable the genetic relationship. In this study, 10 of 20 random primers produced 121 polymorphic bands, with a proportion of polymorphic bands greater than 99%, which could credibly reflect genetic diversity among these isolates.

In this study, the isolates SC10H2, SD and GL, which were identified as *L.* (*SauroLeishmania*) sp. previously [11], had a lower genetic similarity with other strains and first clustered as Clade I. This result from the genome perspective adds to the evidence suggesting the existence of an undescribed *Leishmania* species in China, which is a distinct branch that has low homology with Chinese *L. donovani* strains [9, 33, 34]. In particular, this RAPD result also demonstrated discrimination and differentiation in the relationship among subspecies of *L. donovani* complex. Three isolates, Cy, WenChuan and 801, which were isolated from Gansu, Sichuan and Xinjiang, respectively, were clustered together and separated from other *L. donovani* isolates. This result confirmed that genetic differentiation truly existed in Chinese *L. donovani*. Combined with the results of previous studies [11, 35], it can be concluded that Cy, WenChuan and 801 should be identified as *L. infantum*, which is the causative agent of canine leishmaniasis (CanL) in Sichuan and Gansu. Accordingly, it could be inferred that the VL in Sichuan, Gansu and Xinjiang was caused by *L. infantum*. This conclusion is also in accordance with a previous report [5]. From the UPGMA tree, the isolates KXG-918 and KXG-927 were identified as *L. donovani* in this study, which confirmed again that *L. donovani* was the pathogen of CL in Karamay of Xinjiang. Generally, CL was not the main prevalent type in China, and most were imported. Extensive investigation into indigenous CL in China is needed to obtain solid conclusions regarding the causative agent. In addition, the UPGMA dendrogram showed that *L. donovani* reference strain DD8 did not cluster with KXG-XU, KXG-LIU, 9044, KXG-65 and SC6, which were previously identified as *L. donovani*. This result indicated that there were differences between these Chinese *L. donovani* strains and the *L. donovani* reference strain from India at the genomic level, which was inconsistent with the phylogenetic analysis results obtained with gene markers [8, 34]. As a gene marker only contains partial genome information, and the selective pressure varies among different genes, intraspecific genetic differentiation probably cannot be reflected fully. On the other hand, notable genetic variation is often generated between species or genera with RAPD amplification, so using an individual to represent a species may cause deviation of the phylogenetic results. Consequently, the divergence in this study needs to be further verified by enlarging the sample size or combining it with other methods. In addition, the cluster dendrogram showed that clade B divided into two small branches: Sichuan isolate SC6 from hill foci, Shandong isolate 9044 from plain foci and five other Xinjiang isolates from desert foci. There were still differences among VL isolates from hills, plains and deserts in China, which corroborated the previous report [36]. Although RAPD technology has gradually waned, it is very sensitive for identifying slight intraspecific differences, making it suitable for the differentiation of sibling species.

For the species-specific segments of RAPD, further bioinformatic analysis is beneficial for the exploration of genetic information and the development of specific genetic markers. In this study, three *L. donovani* complex species-specific DNA markers were obtained and preliminarily verified. However, according to their distribution of BLAST hits in NCBI, only the primers of marker 1-AD17 may have a greater specificity for amplification of the *L. donovani* complex. We considered that the differences in primer binding sites or annealing sites of amplification were the cause of the generation of differential DNA fragments of diverse species in RAPD, which was proposed in a previous report [25]. Thus, the SCAR marker 1-AD17 has the potential to be developed into a rapid diagnostic marker of kala-azar, as it was able to separate *L. donovani* and *L. infantum* from the other species in this study. Admittedly, the *L. donovani* complex-specific DNA marker in this study still has certain limits because the species of *Leishmania* are multifarious, and less genomic information is available. Therefore, more parasite samples and patient specimens would be needed to test the specificity.

Through bioinformatic analysis, the three markers were all located on large chromosomes instead of kinetoplasts, which was similar to some other reports [28, 37, 38]. This may be related to the fact that multicopy genes are found preferentially on disomic chromosomes [39], which would increase the probability of random primers binding to them. As genome sequences of different *Leishmania* species are highly conserved [39], the amplification loci of RAPD are frequently located in variable regions. In this study, although there were 4 to 7 ORFs in the three markers, only 3-O13 had two potential promotors, and ORF-4 had the potential to encode proteins. The following protein prediction analysis showed that the hypothetical protein had a higher antigenic index and surface probability. Nevertheless, all of these findings need further experiments for verification.

## Conclusions

Our results verified that the undescribed *Leishmania* species causing VL in China was a unique clade distinguished from *L. donovani* and revealed that there was genetic differentiation among Chinese *L. donovani* isolates at the genome level. Three *L. donovani* complex species-specific DNA markers in 17 available *Leishmania* strains were developed and analysed preliminarily through BLAST and bioinformatics, which may provide a foundation for developing new specific diagnostic markers of VL and performing research on specific gene functions. Nevertheless, the collection of more strains from different origins and patient specimens would be necessary to achieve more accurate intraspecific classification of Chinese *L. donovani* and effective verification of these specific SCAR markers.

## Supplementary Information

**Additional file 1: Table S1.** Genetic similarity matrix among the 17 *Leishmania* strains by NTSYS.

**Additional file 2: Table S2.** Gene sequences of the converted *L. donovani* complex specific SCAR markers.

## Data Availability

The datasets used and analysed during the current study are available from the corresponding author upon reasonable request.

## Abbreviations

RAPD: Random amplified polymorphic DNA
SCAR: sequence characterized amplified regions
VL: visceral leishmaniasis
CanL: canine leishmaniasis
cyt *b*: cytochrome *b*
HSP70: heat shock protein 70
NTSYS: Numerical Taxonomy and Multivariate Analysis System
UPGMA: unweighted pair-group method with arithmetic means
