# Supplementary material for: Genetic diversity analysis of Chinese Leishmania isolates and development of L. donovani complex-specific markers by RAPD
Source: BMC Infect Dis. 2021 May 21;21:464. doi: 10.1186/s12879-021-06163-y (PMC8140445; doi:10.1186/s12879-021-06163-y)
Supplement: Supplementary file 1 — Additional file 1: Table S1. Genetic similarity matrix among the 17 Leishmania strains by NTSYS. [file 12879_2021_6163_MOESM1_ESM.docx]

**Table S1**. Genetic similarity matrix among the 17 *Leishmania* strains by NTSYS.

|  | **1** | **2** | **3** | **4** | **5** | **6** | **7** | **8** | **9** | **10** | **11** | **12** | **13** | **14** | **15** | **16** | **17** |
| --- | --- | --- | --- | --- | --- | --- | --- | --- | --- | --- | --- | --- | --- | --- | --- | --- | --- |
| **1 DD8** | - |  |  |  |  |  |  |  |  |  |  |  |  |  |  |  |  |
| **2 KXG-918** | 0.832 | - |  |  |  |  |  |  |  |  |  |  |  |  |  |  |  |
| **3 KXG-927** | 0.850 | 0.944 | - |  |  |  |  |  |  |  |  |  |  |  |  |  |  |
| **4 GERBILLI** | 0.495 | 0.477 | 0.477 | - |  |  |  |  |  |  |  |  |  |  |  |  |  |
| **5 EJNI-154** | 0.514 | 0.495 | 0.495 | 0.981 | - |  |  |  |  |  |  |  |  |  |  |  |  |
| **6 SD** | 0.486 | 0.505 | 0.467 | 0.505 | 0.505 | - |  |  |  |  |  |  |  |  |  |  |  |
| **7 GL** | 0.477 | 0.495 | 0.458 | 0.495 | 0.495 | 0.991 | - |  |  |  |  |  |  |  |  |  |  |
| **8 9044** | 0.869 | 0.944 | 0.981 | 0.477 | 0.495 | 0.467 | 0.458 | - |  |  |  |  |  |  |  |  |  |
| **9 KXG-XU** | 0.850 | 0.963 | 0.981 | 0.477 | 0.495 | 0.467 | 0.458 | 0.981 | - |  |  |  |  |  |  |  |  |
| **10 SC6** | 0.832 | 0.869 | 0.907 | 0.458 | 0.458 | 0.467 | 0.458 | 0.925 | 0.907 | - |  |  |  |  |  |  |  |
| **11 KXG-65** | 0.860 | 0.972 | 0.972 | 0.486 | 0.505 | 0.477 | 0.467 | 0.972 | 0.991 | 0.897 | - |  |  |  |  |  |  |
| **12 Cy** | 0.888 | 0.813 | 0.813 | 0.514 | 0.533 | 0.505 | 0.495 | 0.832 | 0.832 | 0.794 | 0.841 | - |  |  |  |  |  |
| **13 WenChuan** | 0.888 | 0.813 | 0.813 | 0.514 | 0.533 | 0.505 | 0.495 | 0.832 | 0.832 | 0.794 | 0.841 | 1.000 | - |  |  |  |  |
| **14 801** | 0.888 | 0.813 | 0.813 | 0.514 | 0.533 | 0.505 | 0.495 | 0.832 | 0.832 | 0.794 | 0.841 | 1.000 | 1.000 | - |  |  |  |
| **15 KXG-LIU** | 0.850 | 0.963 | 0.981 | 0.477 | 0.495 | 0.467 | 0.458 | 0.981 | 1.000 | 0.907 | 0.991 | 0.832 | 0.832 | 0.832 | - |  |  |
| **16 K27** | 0.617 | 0.636 | 0.598 | 0.598 | 0.617 | 0.533 | 0.523 | 0.617 | 0.617 | 0.617 | 0.626 | 0.617 | 0.617 | 0.617 | 0.617 | - |  |
| **17 SC10H2** | 0.458 | 0.477 | 0.439 | 0.495 | 0.495 | 0.972 | 0.963 | 0.439 | 0.439 | 0.439 | 0.449 | 0.495 | 0.495 | 0.495 | 0.439 | 0.523 | - |
