# Supplementary material for: Genetic diversity analysis of Chinese Leishmania isolates and development of L. donovani complex-specific markers by RAPD
Source: BMC Infect Dis. 2021 May 21;21:464. doi: 10.1186/s12879-021-06163-y (PMC8140445; doi:10.1186/s12879-021-06163-y)
Supplement: Supplementary file 2 — Additional file 2: Table S2. Gene sequences of the converted L. donovani complex specific SCAR markers. [file 12879_2021_6163_MOESM2_ESM.docx]

**Table S2.** Gene sequences of the converted *L. donovani* complex specific SCAR markers.

| **SCAR markers** | **Gene sequences** |
| --- | --- |
| **Marker1-AD17** | TGGCAAACCCTGTATGAGGAAAACGTTTTTTTTTTTGTTGTTGGCTGTGTGCCCTGTTTGGGCGGTGCGTGAATACAGGGGAAGATGATCACGAACACGCAGCTCTAATCGCGAGAGCATGTGAGTGAGATCTCATCAGTTACGCGACGGCATTCGTCCAGTCGCAAGTCTGCGCGTCAACGGATCACTAAAAATGCCACCTTTCACACATGCGGCGGGCAAGTCGCGTTGGGTCGGTGGCATCAAAAAGTCGTCGCTCACGCCTATGTGCTCCATGGGGCTCTCGCACCCAACGGCCAGACCGCGTTTACGCGTCGGCTTGCGGACGTAGTGATCATAGAACGACGTCGGCCCGTTGGAGAGGCGGTCGACGCACGCCTGCGTCTCGCTGTCGTACTGCGCTTCGTTGATGGGCTGCATGAGGTAGTCGCTGTCCAGCGTTGAGCAAACGCGGTTGTACGCGAGGAAGTTCGACGCCGTTGGTGGGCTATGCAAATCGATGGTGACGCCAACCTCCTTGCAGTCGACGACGACCTTCACGGGTACAGCAGGGGGTTCTGTACTGCTCATGGCCTCGTCTTGACACGCCGGCGTTCCGCGAGAAAGGGAGAAAAGCAACCTATGAGGGTTTGCCAA |
| **Marker2-A816** | GTGACGTAGGCATGCCAGCAAGGTGGCAGGCTAGGAGGAGTTTCGACAGCCGACCTCCTCGAGGTGACGCCACTGGCTTTACTCGCTCGGAAAGCTGCAGAGGAGTCTGGAGGGAGGAAAGACGGGAGGAAGGGACGGGGTCTTGGAGTGGTGACGATGTTGCTGCGACGTATTCTGCAACGCTTGTCAGTGTCCGTTGTCCGCTGCCGGTTGGCCATGTCCGGGCGATGAGTGCGTGAGCGTGTGCTGAACGCGTCTGCCGCTCTGCGTCTCAACGGATGTCAATGGCTGGTCTTTGCACTTTTCCAACCTTCATGCTCTTCACCGCCTCTTTACTCGCGTCCTTTTCTGTTTTCTCTCTTTCGCACTTCTCCTGTAAACGTTTTTGCCCCTCTTCATCCCCACCCCTACGTCAC |
| **Marker3-O13** | GTCAGAGTCCTCGCGGGGTATTCGGTAGCGCAAGAGAGAGCGGAAACGGTCCGGCGTCCAGCGCTGCAAGTGAGCCAGCGTCTTGCGCGGCTGCATACGACACGTACGCGTCGCGCTGGGCGCTCTCCGCGGCAATACTGCGTGTCCAGACACTCCAGCTGCAGGCAAAGTTGGTACCGCCCGGGTGTGTGTCCAAAAACGCGATTGTGGAGGAGATGCCGATACTGTCGGCGAGCTCCACAGAGCTGAGGGTGACGACTGCTGACAGCATCGAAGTCGACGTGTGCTTTGGTGTCGTGCAGGTGCTCGAGTCCATGTGGCGTAACTATCAGCAAAGCCTGCCTTGCGCGCGATATCGAAAGCGTCTGCATCTGCTGCGGCCCACCGGGGGACATGCAACGCATACCCGTGTCCTTCGTCAGGCGTCGGAGCCGAATAGTGTGAGTCTGTCACAGGATGCTGCGACCGCCTGGCGACCATCGGCGCAGCAGAGGTGGTGGTTCGCGTTACGGTGCGTGCTGGACGATGTACGGCAGCATCGGCAGATCTTCGGCTTGCACGAATGGAAACGGTGCGAAGTAGTGGAGGCGATGGTGCAATTCGGGCAGCTGCGTAGAGTCTACACCGAGTACTGGAAGCGCGCCAAGGGGGTCATCTGGGCCCCGCCCTTGAGCGCATTGGAGGAGAAGCGTTTGAAGTCGATGGAGCGGCAGCTGTCATTGTGGCAAGTTATTTATCTGCGGTGCCTTTCTCATGCACAGCTCGTTGTAGAGCAAGAGAGCTATGCGCGGCAGCAGGACTACATTGAAGAGGCCCGCCAGCGCATCAAAAGCGGCTTACTGAGCTCAAGCGGGAACGACGGGGGCGGCAGACCGACATCGTTTCTGTGGGATTGGCTTTTTGGCAGGTCCACACGCAAGAATGATACTGCAACTCTGTCGAGGAAATCGTTGGCTTCCATCGCGTCTCGCGACGGCGTGGGCGTGATATCGGTGCCCACGTACAGAGGCTTTTGCTTTCGCGACTTGGTGGCGCTGGAGTGGGACCTTGGGCGACGCTATACCTCCCCGCACTACGCTCGCTTGGCGCACCCGAGGGCACCACAAAACCGCCTACCCGAGCTGGCAGCGGAGAACAGGTTGTACTTCACGTTGCGTGCGCTTTTTCCGAAGCTTATCGTCCGCATCGATCCTATCTACTGGACTCTGAC |
